# Toward healthy aging in Latin America and the Caribbean: leaving no one behind?

**DOI:** 10.26633/RPSP.2021.113

**Published:** 2021-09-01

**Authors:** Norah C. Keating, Leocadio Rodríguez Mañas, Andrés De Francisco

**Affiliations:** 1 International Association of Gerontology and Geriatrics New York United States of America International Association of Gerontology and Geriatrics, New York, United States of America; 2 University Hospital of Getafe Getafe Spain University Hospital of Getafe, Getafe, Spain.; 3 Pan American Health Organization Washington, DC United States of America Pan American Health Organization, Washington, DC, United States of America.

**Keywords:** Aged, healthy aging, Latin America, Caribbean region

The framework of healthy aging has created a structure for research across the United Nations Decade of Healthy Aging (2021-2030) and for action to address the mission of the Sustainable Development Goals “to leave no one behind” (1). Across the decade, researchers, governments and civil society are urged to develop strategies to identify and address inequities and to foster healthy aging. This agenda requires coordinated effort from researchers in Gerontology and Geriatrics to address the broad set of social and health issues in aging including functional status, social participation, and older adults’ needs in the context of social and health care systems (2).

Healthy aging is dynamic. It reflects processes across the latter part of the life course, influenced by individuals’ mental and physical capacities, their environments and the relationships between them. If there is a ‘good fit’ the outcome is wellbeing (3) and opportunities for older adults across countries and regions to be and to do the things they have reason to value (4). The framework requires us to monitor how health status evolves over time and how health systems can influence health trajectories. There is need to fill knowledge gaps in the supportiveness of family environments; and the extent to which communities have sufficient resources to be age-friendly. The influence of macro issues such as climate change and ageism must be brought into the healthy aging discourse. Indicators of wellbeing that reflect person-environment interfaces need to be developed and used to inform policy and practice interventions to reduce inequities.

The manuscripts in this special issue contribute to the global research agenda for the upcoming decade and make important contributions to the state of knowledge of healthy aging in Latin America and Caribbean. Several papers address functional status and model future functional dependence. They address the components of healthy aging: intrinsic capacities related to physical and mental health; and interactions with key environments including households, communities and broader policies. Together they provide insights into the extent to which older persons in the region can realize their goals.

Martinez et al set the broad context of healthy aging by presenting trends over 30 years in life and healthy life expectancy (5). Their work explores the paradox of substantial increases in life expectancy but smaller increases in healthy life expectancy. Their data show that much of late life illness and disability can be prevented or controlled. González-González and others draw similar conclusions, predicting increased prevalence of dependency in Mexico (6). Gómez et al (7) add evidence from Colombia of predictors of late life dependence, including intrinsic capacity, social environments and physical environments (especially living in rural communities). Three papers propose policy solutions to preventable limitations of late life illness. Morsch et al (8) use case studies in several countries to show the efficacy of interventions to support self-management, stating that providing tools for managing individuals’ health is both an economic and an ethical imperative. Pérez-Zepeda et al (9) illustrate how public health approaches during the COVID-19 pandemic in Mexico and Colombia have had differential impact on mortality among older persons. Alonso Bouzón et al (10) argue the efficiency of adapting proven intervention programs to Latin America in order to reduce frailty, using the example of the ADVANTAGE program that is used in countries across Europe.

Families and social networks of older persons are important contexts for healthy aging. Esteve (11) documents household composition of 23 countries, showing that most people live with children and other relatives. Yet presence of family members does not always result in support. In their research in Mexico, Orozco Rocha et al (12) found reduced prevalence and amount of financial transfers from children to older parents in Mexico over time, concluding that children are unable to reduce inequality among those in lowest income categories. In their research on older people in the high Andes, Parodi et al (13) found that limited mobility was associated with inadequate social support.

Policies and programs are presented by several authors as important in addressing inequalities. In their paper on the situation of older people in Colombia, Tamayo Giraldo et al (14) argue that there is a need for public policy to close the gaps in civic participation and formal employment opportunities in order to increase quality of life for older persons. They identify enhancing income security as one of the main ways to do this. The work from Barrientos (15) also provides data on inequalities across Latin America, noting that approximately 85% of people over age 65 have no income security in contrast to the wealthiest 15% for whom income security rises sharply in later life. Primary care and long-term care systems are also viewed as essential components of policy responses to needs of older persons. González-González et al (6) present a set of indicators of health system responsiveness that can be used to monitor its effectiveness, and Villalobos Dintrans et al (16) stress the rising needs for long term care in the region. They provide guidelines for determining the type of long-term care system that is most appropriate for different countries, stating that countries need to move forward before it is too late.

This collection of papers provides a sound basis on which to build knowledge of aging in Latin America and Caribbean the next decade. There is much to be done to underscore the dividends of longevity and to ensure that its benefits are experienced equitably within and across countries. Evidence of inequalities presented in the manuscripts in this issue, along with further research to be undertaken across the decade, can inform policies and systems to increase income security, access to health services, support to older people and their families and infrastructure for communities left behind because of remote geographies or political instability. Strengthening research on healthy aging in Latin America and Caribbean will indeed move us closer to the goal of leaving no older persons behind.

## Disclaimer.

Authors hold sole responsibility for the views expressed in the manuscript, which may not necessarily reflect the opinion or policy of the RPSP/PAJPH and/or PAHO.
